# Factors Affecting Bone Mineral Density Among Snowy Region Residents in Japan: Analysis Using Multiple Linear Regression and Bayesian Network Model

**DOI:** 10.2196/ijmr.8555

**Published:** 2018-05-22

**Authors:** Teppei Suzuki, Tomoko Shimoda, Noriko Takahashi, Kaori Tsutsumi, Mina Samukawa, Sadako Yoshimura, Katsuhiko Ogasawara

**Affiliations:** ^1^ Faculty of Health Sciences Hokkaido University Sapporo Japan

**Keywords:** health care promotion, Bayesian network, health behavior change

## Abstract

**Background:**

As the onset of osteoporosis leads to reduced activities of daily living and may result in patients being bedridden, efforts to prevent decreased bone density are necessary. Various studies on the relationship between sex, age, nutrients, and exercise habits and bone mineral density have been conducted to date. However, for snowy region residents, the magnitude of influence of various factors affecting bone mineral density and the influence level have not been clarified.

**Objective:**

This study aimed to clarify the degree of influence and factors influencing bone mineral density based on survey results on health conditions and lifestyle habits in heavy snow areas.

**Methods:**

A total of 354 citizens who visited a drugstore in the target area were included in a study that included using the brief-type self-administered diet history questionnaire on lifestyle and exercise habits. Height, weight, body composition, and bone densitometer values were analyzed using multiple regression to calculate their association with bone mineral density. In addition, a Bayesian network model was used to determine the influence level of each factor as a conditional probability.

**Results:**

Multiple regression analysis revealed that age, sex, fracture, and calcium intake significantly influenced bone mineral density. In addition, the result of Bayesian network analysis suggested that age and sex affected bone mineral density, whereas nutrients and exercise habits might not have a direct impact. However, calcium intake and the T-score were significant factors affecting the presence or absence of fracture experiences, suggesting that adequate calcium intake is essential for preventing fractures.

**Conclusions:**

In the multiple regression analysis, age, sex, fracture, and calcium intake were selected as factors; however, in the Bayesian analysis, only age and sex affected bone mineral density while nutrients did not. In addition, the fact that calcium intake and the T-score were shown to affect bone fracture history suggests that calcium intake is an important measure that can prevent bone fractures. Overall, these results suggest that measures such as ensuring a bone fracture–free environment and providing nutritional advice for calcium intake can be effective in preventing bone loss.

## Introduction

### Background

The total population of Japan as of October 1, 2015, was 127.11 million. Seniors aged 65 years and older accounted for 26.7% (33.9 million) of the total population [1]. As the elderly population grows, nursing care problems increase. Approximately half of all individuals requiring nursing care need it for reasons including “weakening due to age,” “joint disease,” and “fractures or falls.” Seniors are particularly prone to falls, which occur at least once annually in approximately 20% of seniors, 5% of which result in fractures. Therefore, fractures and falls should be prevented in a rapidly aging society [2]. Fractures caused by osteoporosis require nursing care and may reduce the quality of life in severe cases due to the patient becoming bedridden. To prevent osteoporosis, it is essential to achieve maximum bone mass while people are still young, and as people age, they should be mindful of their diet and exercise habits to prevent bone loss. Therefore, lifestyle-related factors such as nutrition, exercise, and muscle mass are believed to affect bone density [3].

This study was conducted in Iwamizawa City, located in midwestern Hokkaido, approximately 40 km from Sapporo City, the prefectural capital. One of its most notable characteristics is its heavy snowfall, with a very large average annual snowfall (337 cm), and the deepest single snowfall (133 cm) in 2011. Iwamizawa City experiences more snowfall than Sapporo City and approximately twice or more than that in Kushiro City and Hakodate City, making it the region with the heaviest snowfall in Hokkaido.

In regions with heavy snowfall, residents tend to exercise less and are highly at risk for bone fractures due to the snow- and ice-covered roads. Another characteristic of Iwamizawa City is that daylight hours are shorter than that of other regions in the country. The annual amount of daylight across Japan is 1978 hours compared with 1700 hours in Iwamizawa City. Humans need ultraviolet radiation in order to synthesize most of their vitamin D. The primary action of vitamin D is the promotion of calcium absorption in the small intestine, and reduced vitamin D activity inhibits calcium absorption. Persistently low calcium levels can impair bone calcification and reduce bone density [4]. In a previous study conducted in Iwamizawa City, Shimoda et al [5] investigated the body composition and nutritional intake of elderly residents in regions with heavy snowfall. They found that the basal metabolic rate and muscle mass decreased with age, although calories and nutritional intake remained sufficient, suggesting a limited effect of diet.

Last, Iwamizawa City has a high population aging rate at 32.4% as of January 1, 2016; a value approximately 6% higher than that of the whole country. Therefore, it is considered a geographical region with many residents suspected of having osteoporosis [6].

In earlier studies on osteoporosis in the region, Uchida et al [7] reported that measures preventing bone fractures are important in a society with an increasing number of bedridden patients and that maximum bone mass, which peaks between 20 and 30 years, is greatly affected by genetics. Therefore, Uchida et al investigated the factors affecting bone mineral density and found that environmental factors such as lifestyle were strongly associated with bone density. They proposed that studying the relationship between bone density and environmental factors such as nutrition is important from the standpoint of prophylactic medicine. Toda et al [8] conducted a study involving 654 healthy Japanese women aged 40 to 60 years to investigate the lumbar bone mineral density, anthropometric measurements, and 3-day meal, and a survey was also conducted to determine the effects of diet and physical activity on bone density, with the purpose of preventing osteoporosis. The results showed that appropriate exercise and a well-balanced diet were important factors in preventing osteoporosis. However, previous studies did not target regions with heavy snowfall nor did they provide details on nutritional intake. In addition, they did not assess the effects of various nutrients on bone density or whether an influence level existed.

### Objectives

This study aimed to determine how nutritional intake, lifestyle and exercise habits, and body composition information affect bone density in heavy snowfall/cold regions and assess the extent of the effect on the related factors.

## Methods

### Participants and Data Collection

The data used in our study were obtained from a study conducted by a Center of Innovation program entitled, “Study of health status for the purpose of health innovation in Iwamizawa City.” A total of 354 individuals (87 men and 267 women) who visited the city drugstore participated in the study (Table 1).

### Measurement

The survey activities included completing a questionnaire related to lifestyle and exercise habits, completing the brief, self-administered diet history questionnaire so that daily nutritional intake could be determined, and measuring the body composition and bone density. In the nutrition survey, the brief-type self-administered dietary history questionnaire (BDHQ 58-item fixed-portion-type questionnaire) was used [9]. The equipment used consisted of an ultrasonic calcaneus measurement device, A-1000 EXPII (GE Healthcare Japan), for bone density measurements and a WELL-SCAN 900 (Canon Inc) for body composition measurements.

### Data Analysis

Factors that largely affect bone density that were extracted as explanatory variables for the study analysis were bone fracture history; moderate exercise (such as sports or recreation that increase the heart rate for approximately 10 minutes); calcium, vitamin D, and cholesterol intakes per day (through meal); sex; age; body mass index; muscle mass; and degree of obesity (Table 2). A multiple regression analysis using bone density (the T-score) as the criterion variable was performed to analyze the effects of each explanatory variable on bone density. Statistical analyses were performed using JMP Pro version 12.2.0 (SAS Institute Inc).

Next, a Bayesian network was established to determine the influence level between bone density and each explanatory variable. It is a means of expressing the influence level of a target event as a graph model. Bayesian networks are expressed as noncircular directed graphs with directionality links that are interpreted as the direction of the causes and effects of events. The influence level between events is obtained by the conditional probability of a linked root event. Conditional probability is generally expressed as P(A|B) and represents the probability of event A occurring given that event B has occurred. A Bayesian network is a technology frequently used in the field of medicine. Velikova et al [10] designed a Bayesian network model that accounts for the changes of preeclampsia, a type of pregnancy-related disorder, for a certain period of time, and they also proposed a system that supported decision making at the individual level [10-13].

In this study, a Bayesian network was established using bone density (T-score) and explanatory variables extracted from a multiple regression analysis. Table 3 shows the criteria for the T-score. The influence level between each explanatory variable and bone density was determined, the conditional probability (prior probability) was calculated, and the factors affecting bone density status were analyzed. In addition, a sensitivity analysis, in which the parameters of each factor were changed to obtain the posterior probability, was performed, and the combination with the highest probability of osteoporosis or osteopenia occurrence was studied. The variables used in the multiple regression analysis were also used as factors in the Bayesian network model. The Bayesian network was established using BAYOLINK version 7.0.0 (NTT DATA Mathematical Systems Inc).

**Table 1:** Characteristics of study population by sex and age.

Characteristics		n (%)
**Sex**		
	Women	267 (75.4)
	Men	87 (24.6)
**Age, years**		
	20-29	12 (3.4)
	30-39	26 (7.3)
	40-49	24 (6.8)
	50-59	69 (19.5)
	60-69	136 (38.4)
	70-79	76 (21.5)
	80+	11 (3.1)

**Table 2:** Variables with their values as used in the Baysian network model.

Factors	Survey method and contents	Values and ranges
Fracture	Questionnaire: fracture experience	No, yes
Exercise	Questionnaire: medium strength sports that last ≥10 min	No, yes
Calcium	Brief-type dietary history questionnaire	Adequate, slightly inadequate^a^, inadequate^a^
Vitamin D	Brief-type dietary history questionnaire	Adequate, slightly adequate^a^, inadequate^a^
Cholesterol	Brief-type dietary history questionnaire	Adequate, slightly high^a^, high^a^
Sex	Body composition meter	Female, male
Age, years	Body composition meter	<65, ≥65^a^
BMI^c^, kg/m^2^	Body composition meter	Normal (<25), obesity^a^ (≥25)
Muscle mass, kg	Body composition meter	<37.0, 37-44.9, ≥45.0^a^
Obesity, %	Body composition meter	Underweight (<–10), normal (–10 to 10), overweight^a^ (10 to 20), obesity^a^ (≤20)

^a^Risk factor.

^b^BMI: body mass index.

**Table 3:** Criteria for bone density status using T-score.

Bone density status	T-score
Normal	–1.0 and higher
Osteopenia	–1.0 to –2.5
Osteoporosis	–2.5 and lower

**Figure 1:**
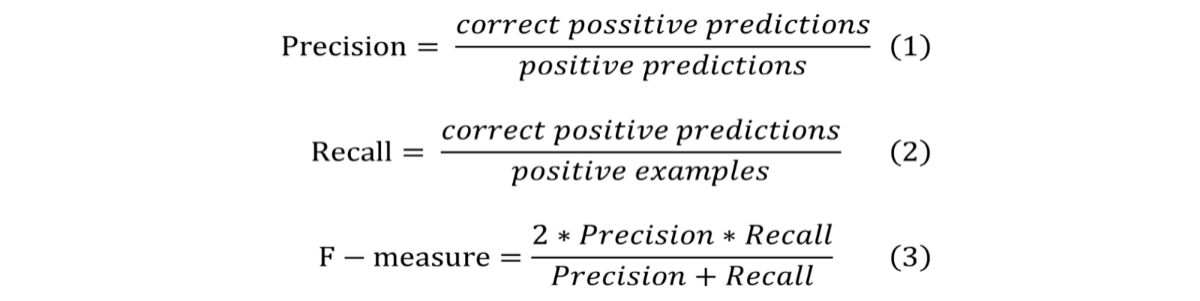
Formulas of precision, recall and F-measure.

Precision, recall, and F-measure were used as indicators to evaluate the results of the Bayesian network. Precision represents data that match the verification value among the data predicted as the target state by reasoning. Recall represents the proportion of data coincident with the estimated value among the target state in the verification data. F-measure represents the harmonic mean of precision and recall.

Mutual information amount represents the changes in the amount of model entropy before and after inputting the observation. As the mutual information amount increases, the influence on bone density, which is the objective variable, increases (Figure 1).

### Ethical Considerations

This study was conducted with the approval of the Ethics Review Committee of the Graduate School of Health Sciences, Hokkaido University (approval number 15-96). For subject recruitment, we presented government-distributed public magazines and information on research cooperation at the drugstore where the survey was conducted. We also explained that participation in the research was voluntary and that there were no disadvantages in declining the participation midway, and we obtained written consent. Data obtained were not used for any purpose other than research and were stored carefully.

## Results

Table 4 shows the participants’ bone density status based on sex, revealing that 43 men had normal bone density, 36 had suspected osteopenia, and 8 had suspected osteoporosis, while 117 women had normal bone density, 112 had suspected osteopenia, and 38 had suspected osteoporosis. Based on these results, approximately half of both men and women were healthy, and the other half were suspected to have osteopenia or osteoporosis Table 5.

Table 6 shows the results of multiple regression analysis. The fewer fractures men had when they were young and the higher their calcium intake, the higher their bone density tended to be. Nutrition, exercise habits, and muscle mass, which have been thought to affect bone density, were not selected for the model.

**Figure 2:**
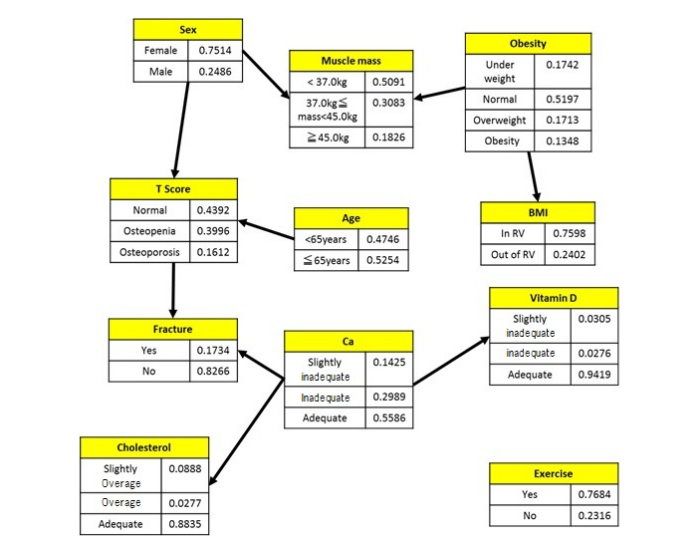
Prior probability by Bayesian network.

Figure 2 shows the Bayesian network in determining the influence level between bone density and each associated factor. The Bayesian network model expresses random variables as nodes and links to nodes with probabilistic dependencies. In the result shown in Figure 2, the T-score represents the state of bone density depending on sex and age. The T-score also shows its direct influence on the presence or absence of fracture. For the value of each factor, the prior probability is shown. The influence level between exercise or muscle mass and the T-score could not be confirmed. In addition, regarding vitamin D, although an influence level with calcium intake was confirmed, a direct influence level with the T-score cannot be confirmed.

**Figure 3:**
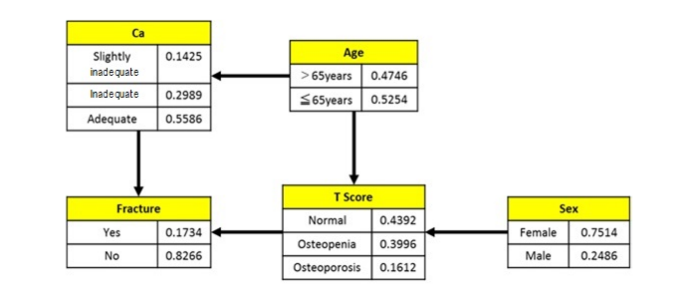
Bayesian network model of factors influencing bone density.

Figure 3 shows a Bayesian network model limited to the effect on bone density using variables extracted from the results of multiple regression analysis. The T-score had an influence level with sex and age and has been affected by the history of bone fracture. In addition, an influence level between calcium intake and age was newly discovered.

Table 7 shows the results of estimated accuracy validation of the model, with the T-score as the criterion variable. When bone density was normal, prediction accuracy was highest. In the case of osteoporosis, prediction accuracy was lowest. Table 8 shows the mutual information of each variable, with the T-score as the criterion variable. Since the mutual information of explanatory variable “age” is the largest, the result that age has the largest influence on the state of bone density was obtained.

**Table 4:** Measurement results.

Characteristics		Male, n	Female, n	Total, n
**Body mass index, kg/m^2^**				
	Normal (<25)	57	213	270
	Obesity (≥25)	28	42	70
**Muscle mass, kg**				
	<37	2	180	182
	37 to 45	28	69	97
	≥45	55	6	61
**Obesity, %**				
	Underweight (<–10)	8	60	68
	Normal (–10 to 10)	43	138	181
	Overweight (10 to 20)	15	31	46
	Obesity (≥20)	19	26	45
**Calcium**				
	Adequate	46	141	187
	Slightly inadequate	9	40	49
	Inadequate	32	77	109
**Vitamin D**				
	Adequate	84	244	328
	Slightly inadequate	0	9	9
	Inadequate	3	5	8
**Cholesterol**				
	Adequate	79	233	312
	Slightly high	4	22	26
	High	4	3	7
**Fracture**				
	Yes	17	32	49
	No	65	215	280
**Exercise**				
	Yes	76	219	295
	No	6	28	34

**Table 5:** Bone density by sex (χ^2^=1.742, *P*=.418).

Bone density	Male (n=87), n (%)	Female (n=267), n (%)
Normal	43 (49)	117 (44.8)
Osteopenia	36 (41)	112 (41.9)
Osteoporosis	8 (9)	38 (14.2)

**Table 6:** Result of multiple regression analysis.

Variable	Regression coefficient	*P* value
Age	–0.67	<.001
Sex	–12.44	<.001
Fracture	–1.55	.009
Calcium	0.01	.027

**Table 7:** T-score prediction accuracy.

Bone density	Precision	Recall	F-measure
Normal	0.56	0.68	0.61
Osteopenia	0.48	0.49	0.48
Osteoporosis	0.5	0.14	0.22

**Table 8:** Mutual information.

Variable	Mutual information
Age	0.065
Fracture	0.014
Sex	0.008
Calcium	0.002

## Discussion

### Multiple Regression Analysis Results

The explanatory variables selected for the model were age, sex, history of bone fracture, and calcium intake, but only age and sex were significant and are not something that an individual can control. However, in relation to bone fracture prevention and calcium intake, bone density can be increased by changing one’s awareness of daily lifestyle factors. According to Kubota et al [14], who studied approaches in preventing bone fracture and osteoporosis through calcium intake, one method to increase calcium intake is to take calcium supplements or consume calcium through food, which was proven to effectively reduce bone fracture risk due to osteoporosis in elderly populations. The degree to which bone density reduction can be prevented by supplements or calcium intake must be studied in the future.

Based on the results of this study, sex and age greatly affect bone density, and factors such as vitamin D and exercise habits, believed to affect bone density, were not included in the analysis. Kubota et al [14] reported that the coadministration of calcium and vitamin D in elderly men and women prevented bone loss in the femoral neck and throughout the whole body. In addition, a study by Matsubara et al [15] suggested the possibility that the rate of bone mineral loss throughout the whole body could be prevented in menopausal women. Therefore, physical exercise and nutrients such as vitamin D must be incorporated into the model, and the results obtained in the analysis must be considered.

### Bayesian Network Analysis Results

The influence level between each factor was determined based on the results obtained through Bayesian network analysis, which are shown in Figures 2 and 3. These results showed that only age and sex affected bone density, and calcium intake and the T-score affected the occurrence of bone fractures; however, calcium intake was the most important measure that can be implemented to prevent bone fractures.

**Figure 4:**
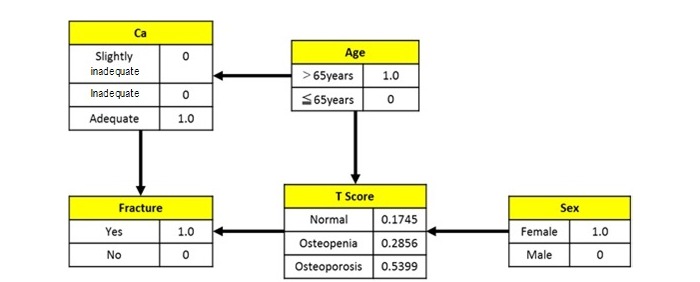
The combination with the highest probability of osteoporosis.

**Figure 5:**
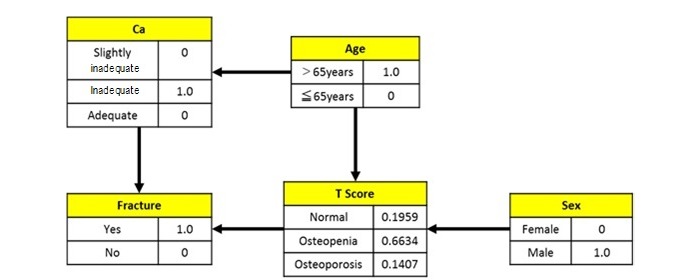
The combination with the highest probability of osteopenia.

A sensitivity analysis performed on the prior probability obtained in the Bayesian network analysis revealed that several factors contributed to the highest probability of developing osteoporosis and osteopenia, as shown in Figures 4 and 5.

**Figure 6:**
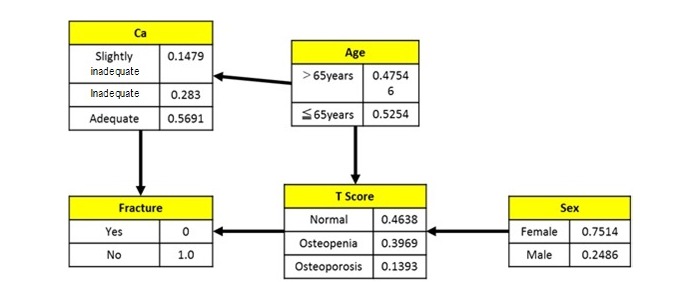
Posterior probability of calcium and T-score in the absence of fracture experience.

The combination with the highest probability to develop osteoporosis consisted of women aged 65 years and older with appropriate calcium intake and history of bone fracture, with the posterior probability of 0.5399 (3.35 times the prior probability). The combination with the highest probability to develop osteopenia consisted of men aged 65 years and older with insufficient calcium intake and history of bone fracture, with the posterior probability of 0.6634 (1.66 times the prior probability). Both results showed that the posterior probability increased when an individual had a history of bone fracture and was aged 65 years or older. However, in terms of sex differences, the results suggested that women had a higher probability of developing osteoporosis while men had a higher probability of developing osteopenia. In addition, the probability of developing osteopenia is suspected to increase when calcium intake is insufficient, as shown in Figure 5. Therefore, particularly in men aged 65 years and older, bone loss can be prevented by a periodic examination of calcium intake and providing nutritional advice.

The mutual information in Table 8 shows that age has the highest level of effect on bone density, followed by bone fracture history, sex, and calcium intake. Age and sex are uncontrollable factors. However, because bone fracture history and calcium intake can be controlled, bone loss can be prevented through measures such as ensuring a safe environment in which bone fracture does not occur and providing nutritional advice, including calcium intake.

Bayesian networks are also believed to be useful in establishing health care policies in the region by changing the posterior probability of each factor in the Bayesian network model and obtaining the factors associated with those changes. Miyauchi et al [16,17] established a Bayesian network using the results of a specific medical checkup and created an evaluation system for the specified medical checkup focusing on lifestyle-related diseases such as metabolic syndrome. As an example, a Bayesian network for the response “I have no history of bone fracture” is shown in Figure 6, with age and sex distributions set to the same conditions as in Figure 3, and the changes in the posterior probabilities are shown in Tables 9 and 10. Factors affecting bone fracture history are calcium intake and T-score. The percentage of individuals deficient in calcium intake should be decreased by 0.54%, and the percentage of persons with appropriate calcium intake must increase by 1.05%. The percentage of individuals within the appropriate T-score range must also increase by 2.46%, those with osteopenia must decrease by 0.27%, and those with osteoporosis must decrease by 2.19%. These methods are believed to be useful indices in establishing health care policies in the region.

### Limitations and Future Research Directions

This study has some limitations. First, its reliability is possibly low because data on lifestyle and exercise habits were self-reported. To address this, an activity meter must be distributed to the participants to determine whether exercise was performed definitively. In addition, for items related to exercise in this survey, moderate exercise was defined as “sports or recreation that increases the heart rate for approximately 10 minutes.” However, the frequency and duration of exercise that effectively prevented bone loss are presently unknown. In the future, a more detailed survey must be conducted to determine the appropriate frequency and duration of exercise required to prevent bone loss.

Another limitation was the possibility that the amount of data for our study results was relatively small. In a cohort study conducted in Hisayama, Yoshida et al [18] analyzed data of 1550 participants aged 65 years and older to determine the cause of functional impairment in the elderly people. Kishimoto et al [19] conducted a study on 803 patients to determine their risk of cognitive impairment. Similarly, by continuing our study to gather more data for analysis, we believe that the analytical accuracy of our study can be improved. Table 7 shows the estimated accuracy of osteoporosis, in particular, to be greatly reduced. This reduction in accuracy resulted from a small sample size since only 46 individuals had osteoporosis out of 354 study participants. In order to collect more data, more residents should participate in the study through proactive activities in the region such as establishing a mechanism where participants can see a personal benefit in participating. In addition, an environment that is easily accessible for residents to participate and want to participate should be established such as providing them with incentives for study participation.

In this study, we conducted a survey on customers who came to the drugstore. Therefore, we think that the health condition of customers based on this drugstore may have an impact on the survey results. In the future, public facilities should be used as a survey site, and surveys should be conducted in a wide range of participants. This study covered a heavily snowy area, and many western residents have restricted activities. In addition, the arable land area ratio (arable land area/total land area) of the city is 41.2%, with many residents involved in agriculture.

**Table 9:** Changes in the posterior probability of calcium in the absence of fracture experience.

Calcium	Prior probability	Posterior probability	Variation (%)
Inadequate	0.30	0.28	–1.59
Slightly inadequate	0.14	0.15	0.54
Adequate	0.56	0.57	–1.05

**Table 10:** Changes in posterior probability of T-score in the absence of fracture experience.

T-score	Prior probability	Posterior probability	Variation (%)
Normal	0.44	0.46	2.46
Osteopenia	0.4	0.40	0.27
Osteoporosis	0.16	0.14	2.19

However, as farm work is limited during winter season compared with summer season, opportunities to move the body are expectedly reduced [19].

In this study, since the survey was conducted in winter, comparison with the summer season has not been done. Therefore, the influencing factors on the health condition should be analyzed by collecting data in summer and comparing it with the data obtained in winter.

### Conclusion

Based on the multiple regression analysis results, age, sex, history of bone fracture, and calcium intake were selected as models, but the effects of other nutrients and exercise were not assessed. In the Bayesian analysis results, age and sex were the only factors that affected bone density; nutrients or exercise did not have an effect. However, the fact that calcium intake and T-score were shown to affect bone fracture history suggests that calcium intake is an important measure to prevent bone fractures. Overall, these results suggest that measures such as ensuring a safe environment to prevent bone fractures and providing nutritional advice for calcium intake are effective in preventing bone loss.

## Abbreviations

BDHQ: brief-type dietary history questionnaire
BMI: body mass index
